# An atypical presentation of cystic fibrosis: a case report

**DOI:** 10.1186/1752-1947-2-201

**Published:** 2008-06-12

**Authors:** Deepak Joshi, Anil Dhawan, Alistair J Baker, Michael A Heneghan

**Affiliations:** 1 Institute of Liver Studies, Kings College Hospital, London SE5 9RS, UK; 2 Institute of Liver Studies (Paediatrics), Kings College Hospital, London SE5 9RS, UK

## Abstract

**Introduction:**

The presentation of cystic fibrosis is dependant upon which organs are affected. Common presentations include chronic respiratory infections and malabsorption. Patients with atypical disease tend to present late in childhood or as adults. Eye manifestations of cystic fibrosis are less well known.

**Case presentation:**

A 14-year-old Caucasian boy presented with tiredness and difficulty seeing at night, over a period of 6 months. Good vision was only described in bright conditions. There was no history of jaundice, steatorrhea or diarrhoea.

**Conclusion:**

This is the first reported case of newly diagnosed cystic fibrosis-related liver disease in a teenage boy, whose presenting symptom was night blindness secondary to vitamin A deficiency.

## Introduction

Cystic fibrosis (CF) is the most common autosomal recessive disease in Northern Europe. This case highlights the broad spectrum of presentation of CF. Although the respiratory and gastro-intestinal symptoms are well recognised, the eye manifestations of CF are less well known. We describe a primary presentation of CF-related liver disease at age 14 in a boy presenting with night blindness secondary to vitamin A deficiency.

## Case presentation

A 14-year-old Caucasian boy presented with tiredness and difficulty seeing at night, over a period of 6 months. He described good vision only in bright conditions. No past medical history was noted, but he did describe intermittent abdominal discomfort. There was no history of jaundice, steatorrhea or diarrhoea. The patient had presented to his general practitioner with the same symptoms and had been empirically commenced on oral vitamin A supplements, before being referred to local hospital services. Clinical examination revealed clubbing of the hands and feet, and gynaecomastia. His liver was not tender but palpable 3 cm below the right costal margin. Respiratory examination was unremarkable. Eye examination revealed bilateral Bitot's spots. Serological evaluation revealed an albumin of 22 g/l (normal range (NR) = 35 to 50 g/l), bilirubin of 34 μmol/l (NR = 0 to 17 μmol/l), alkaline phosphatase of 693 IU/l (NR = 116 to 483 IU/l), aspartate aminotransferase of 112 IU/l (NR = 1 to 50 IU/L), gamma-glutamyltranspeptidase of 158 IU/l (NR = 1 to 55 IU/l), and an international normalised ratio of 1.32 (NR = 0.9 to 1.2). Serum vitamin A levels were at the lower end of normal, at 0.32 μmol/l (NR = 0.3 to 4.5 μmol/l). Tests for pancreatic endocrine and exocrine function were normal.

A chest radiograph revealed bronchial wall thickening. An ultrasound scan of the liver demonstrated an enlarged left lobe, with reversed flow within the portal vein and an enlarged spleen (15.7 cm). Hepatitis B and C virus serology was negative. Sweat tests were positive on two separate occasions with measured sweat chloride of 123 mmol/l (NR = 0 to 50 mmol/l). Genetic analysis identified the ΔF508 and G542X mutations. Liver biopsy showed fatty and fibrotic liver tissue with mild portal and focal perisinusoidal fibrosis with spared areas of focal cirrhosis, consistent with CF-related liver disease. A diagnosis of CF-related liver disease presenting with severe vitamin A deficiency was made. The symptoms of night blindness improved subsequently and the patient is currently active on the liver transplantation list.

## Discussion

The spectrum of clinical presentation in CF is vast, and depends on which organs are affected. Patients may present with typical CF-related symptoms such as chronic respiratory infections or malabsorption. Patients with atypical disease tend to present late in childhood or as adults with less widely known complications such as pancreatitis, congenital absence of the vas deferens and azoospermia, or nasal polyps [1]. This is the first reported case of CF in a teenage boy with newly diagnosed CF-related liver disease, whose presenting symptom was night blindness secondary to vitamin A deficiency. Although serum vitamin A levels were within normal ranges, the patient had been commenced on replacement therapy, and eye signs were evident on examination consistent with chronically low vitamin A levels.

Vitamin A deficiency in CF may be caused by a variety of mechanisms: pancreatic insufficiency and reduced entero-hepatic circulation of bile acids leading to malabsorption of fat soluble vitamins (A, D, E, K), and reduced concentrations of retinol binding protein, essential for transport of retinol from the liver to tissues. Eye symptoms in CF are well documented. These include xerophthalmia, papilloedema and retinal haemorrhages. Xerophthalmia is common, and thought to be a primary manifestation of CF [2]. Vitamin A deficiency in developed countries usually occurs in conjunction with malabsorption states such as CF, pancreatic insufficiency and liver disease. It is common in CF [3] but often subclinical. Night blindness (nyctalopia) is the most common and earliest symptom of vitamin A deficiency [4]. Bitot's spots, triangular, perilimbal grey plaques of keratinized conjunctival debris, and xerosis, dry granular patches, tend to occur after more prolonged periods of deficiency. Early ocular changes are reversible with adequate replacement, whilst late changes result in permanent corneal damage and visual loss [5].

## Conclusion

The spectrum of presentation of CF is wide and varied, and this case highlights an atypical presentation. It is therefore important to consider the diagnosis of CF in adolescents and young adults who present with night blindness and vitamin A deficiency.

## Abbreviations

CF: cystic fibrosis; NR: normal range.

## Competing interests

The authors declare that they have no competing interests.

## Authors' contributions

DJ was involved in the writing of the case report, AD, AJB and MAH were involved in the reviewing of the article. All authors were involved in the patient's care. All authors read and approved the final manuscript.

## Consent

Written informed consent was obtained from the parents of the patient for publication of this case report and accompanying images. A copy of the written consent is available for review by the Editor-in-Chief of this journal.
